# Understanding the Impact of Schwartz Rounds on Pediatric Clinicians' Well-Being Using the Positive Emotion, Engagement, Relationships, Meaning, and Accomplishment (PERMA) Model for Flourishing: A Qualitative Analysis

**DOI:** 10.7759/cureus.46324

**Published:** 2023-10-01

**Authors:** Uchechi Oddiri, Shahriar Islam, Wei-Hsin Lu

**Affiliations:** 1 Department of Pediatrics, Renaissance School of Medicine at Stony Brook University, Stony Brook, USA; 2 Department of Family, Population, and Preventative Medicine, Renaissance School of Medicine at Stony Brook University, Stony Brook, USA

**Keywords:** healthcare provider, clinician, positive psychology, perma, schwartz rounds, flourishing, well-being

## Abstract

Objective

Schwartz Rounds (SR) is an interdisciplinary, case-based forum that augments compassionate care at the bedside and promotes discussion of the psycho-emotional aspects of patient care. This pilot study analyzed the perspectives of pediatric SR attendees through the lens of *human flourishing*, as defined by “PERMA” (positive emotion, engagement, relationships, meaning, and accomplishment).

Methods

This qualitative study was conducted at our Children’s Hospital from September 2023 to October 2023. Focus group questions were developed from the Secure Flourishing Index and Community Workplace Flourishing validated tools. Clinicians who attended at least one SR in our Children’s Hospital from January 2019 through September 2021 were recruited to participate in focus groups. Transcripts were analyzed using a direct content analysis based on the PERMA framework. Coded content from participants who attended >2 SR since 2019 was considered frequent (FR), whereas that of participants who attended ≤2 SR since 2019 was considered non-frequent (NFR).

Results

Sixteen clinicians (14 pediatric and two non-pediatric) participated in focus groups, including seven FR and nine NFR participants. There were nine emerging themes, eight of which were characterized among frequent SR attendees: SR serves as a safe and trusted space, promotes validation and support, facilitates introspective thinking, stimulates perspective shifts, augments compassion, reaffirms purpose, positively impacts one’s professional identity, and no impact on resilience. In comparison, five of these themes and another theme (humanizes medicine) were identified among non-frequent attendees. All themes reflected one or more PERMA categories.

Conclusion

SR has the potential to augment human flourishing and holds a vital role in promoting a supportive environment in the workplace. SR thereby offers institutions an effective interventional tool to promote elevated well-being in the workforce.

## Introduction

Schwartz Rounds (SR) is an interdisciplinary forum for clinicians to discuss the psychosocial and emotional issues that arise in patient care, with the purpose of augmenting compassionate care [1]. SR was created in the 1990s by the Schwartz Center for Compassionate Healthcare in honor of Ken Schwartz, an American lawyer who was diagnosed with advanced lung cancer in his 40s, succumbing to his illness 10 months after his diagnosis. Schwartz emphasized how “the smallest acts of kindness” make “the unbearable bearable,” and founded the Schwartz Center to “ensure all patients receive compassionate and humane care” [2]. In today’s healthcare landscape, SR can promote and foster an empathetic, patient-centered approach to patient care amidst the evolving complexities of modern medicine.

Over 450 healthcare organizations in the United States, United Kingdom, Ireland, Australia, New Zealand, and Canada have implemented SR since its inception in 2002 [3]. These one-hour meetings occur on a monthly or bi-monthly basis and are theme-specific. They commence with a panel of approximately three clinicians who center the discussion on a patient case and highlight their own emotional challenges while caring for the patient. The clinicians then open the discussion to the assembled peer group, which can vary in size from several dozen to hundreds depending on the availability of SR attendees. The peer group is invited to respond empathically and non-judgmentally and share their own anecdotal experiences as they pertain to the theme.

The benefits of SR are vast as they allow for opportunities to normalize and validate emotional experiences [4], increase understanding between staff [5], and build an appreciation of the roles and contributions of colleagues [6]. Dawson et al. demonstrated that SR benefits clinicians’ well-being by reducing psychological distress as they can process aspects of their experience that would otherwise be ignored or left repressed [7]. Furthermore, Lown and Manning observed that SR attendees experienced decreased perceived stress and increased ability to manage the psychosocial and emotional demands of patient care [6]. While the advantages of SR have been evidenced by numerous empirical studies [5-9], no study to date has provided a thematic analysis of the experiences of SR attendees as it relates to human flourishing.

The theory of *human flourishing* or “flourishing,” conceived by Martin Seligman is a conception of well-being that helps to reimagine health and wellness more broadly than existing measures [10]. In his theory, well-being and flourishing are interchangeable but must be viewed as more than happiness and life satisfaction. Flourishing is multifaceted, buildable, and optimized by the five components of “PERMA”: *positive emotions* (the experience of positive feelings such as pleasure, comfort, and joy), *engagement* (being absorbed in an activity), *relationships* (cultivating meaningful interactions with others), *meaning* (fostering a connection to something greater than the self), and *accomplishment* (the fulfillment of goals) [10]. The promotion of these elements has been associated with decreased occupational burnout [11] and enhanced individual well-being [12] and can be applied to interventions that are geared toward improving well-being and reducing burnout in clinicians [13,14].

The main objective of this qualitative pilot study was to assess SR’s potential for enhancing clinician flourishing. Using considerable insight gathered from clinicians’ self-reported experiences in attending SR, this study contributes to the ongoing conversation on how SR impacts clinicians’ well-being.

This article was previously presented as a “Flash Talk” at the Annual Academic Pediatric Association Regions 2 & 3 Meeting on March 25, 2022, as a poster at the 2022 International Conference on Physician Health on October 14, 2022, and as an oral abstract presentation at the 2023 Northeast Group on Educational Affairs Annual Conference on April 15, 2023.

## Materials and methods

Study design

Our tertiary care Children’s Hospital is our county’s only Level III Neonatal Intensive Care Unit and Level I Pediatric Trauma Center. We have been conducting SR since early 2015. With grounded theory qualitative methodology, this study used data collected from focus groups (FG) and interviews conducted with clinicians who participated in SR to obtain a comprehensive and in-depth understanding of clinicians’ perceptions of the impact of SR on their well-being. After a brief pause due to the COVID-19 pandemic from March 2020 to October 2020, SR resumed in the fall of 2020 but underwent another hiatus from December 2020 to April 2021. Clinicians who attended at least one SR from January 2019 through September 2021 were recruited via email to participate in a 90-minute FG. We only invited SR attendees who participated in SR since 2019 to limit recall bias. Study participants were compensated with a $10 gift card. This study was approved by the Institutional Review Board (IRB#2020-00560).

Data collection and analysis

FG were led by the same facilitator (UO) using a semi-structured FG protocol (supplemental table in appendices). FG questions were developed from the Secure Flourishing Index [15] and Community Workplace Flourishing [16] tools, which assess and measure one’s personal and workplace flourishing, respectively. FG were audio recorded and transcribed verbatim. All study investigators independently analyzed all FG transcripts. In the FG transcripts, a sentence or phrase served as the unit of analysis, and direct content analysis was performed to inductively identify and develop emergent and salient themes that aligned with the PERMA framework [17]. Subsequently, the researchers met to compare coded content and arrived at a consensus on key themes after extensive discussion and repeated review of the original transcripts to ensure the analysis remained grounded by participants’ statements. Coded content from participants who attended >2 SR since 2019 was considered frequent (FR) participants, whereas that of participants who attended ≤2 SR since 2019 was considered non-frequent (NFR). Verbatim quotations from the discussion were chosen to provide support for the analysis.

In these discussions, we recognized that having a diverse research team, which included investigators of various subject familiarity, ensured reflexivity. Peer debriefing and reflexivity were performed to establish greater objectivity in this study’s analysis.

## Results

From September to October 2021, five FG and one 1-on-1 interview were conducted. Sessions ranged between 22 and 83 minutes in length, and data saturation was determined after six sessions. FG participants consisted of a total of sixteen clinicians from various disciplines (Table 1), including seven FR and nine NFR participants.

**Table 1:** Demographics of Focus Group Participants FR: Frequent Schwartz Rounds attendees; ID: Identification number; NFR: Non-frequent Schwartz Rounds attendees; SR: Schwartz Rounds.

ID	Gender	Job Title	Job Specialty	# of SR Attended
Frequent Attendees (attended >2 SR sessions since 2019, n=7)				
FR1	Female	Nurse	Pediatric Subspecialist	7
FR2	Female	Nurse Practitioner	Pediatric Subspecialist	3
FR3	Female	Attending	Pediatric Subspecialist	4+
FR4	Male	Attending	Pediatric Subspecialist	7+
FR5	Female	Attending	Pediatric Subspecialist	4
FR6	Female	Nurse Practitioner	Pediatric Subspecialist	3
FR7	Female	Nurse Practitioner	Psychiatry	3
Non-frequent Attendees (attended ≤2 SR sessions since 2019, n=9)				
NFR1	Female	Attending	Pediatric Subspecialist	2
NFR2	Female	Attending	Pediatrics	1
NFR3	Female	Nurse	Pediatric Subspecialist	1
NFR4	Female	Nurse	OB/GYN	1
NFR5	Female	Attending	Pediatrics	1
NFR6	Female	Chief Resident	Pediatrics	1
NFR7	Male	Resident	Pediatrics	2
NFR8	Female	Respiratory Therapist	Pediatrics	1
NFR9	Female	Nurse Practitioner	Pediatric Subspecialist	1

FG participants were initially assessed through general questions about their experiences in clinical work, patient engagement, colleague engagement, and emotional well-being (Table 2).

**Table 2:** Focus Group Themes From General Questions Focus group themes from general questions about focus group participants’ clinical work experience, patient engagement, colleague engagement, and emotional well-being.

Category	Themes
Clinical Work	(1) Meaningful, gratifying, and rewarding; (2) Privilege to work as a healthcare professional; (3) Busy, taxing, and draining some days; (4) Challenges with COVID-19
Patient Engagement	(1) Most rewarding and meaningful part of the job; (2) Time constraints is a barrier to patient engagement; (3) Unique challenges to patient engagement due to COVID-19 (no physical contact, mask wearing, etc.)
Colleague Engagement	(1) Collegial, friendly, respectful, and collaborative; (2) Engaging with colleagues can “refill one’s emotional bucket”; (3) COVID-19 fuels burnout and conflict between colleagues
Emotional Well-being	(1) Importance of work-life balance; (2) Prioritizing others’ well-being over their own; (3) Stress and anxiety due to work-related issues and unpredictability of COVID-19; (4) Partake in hobbies, mindfulness, and meditation to maintain well-being

**Figure 1:**
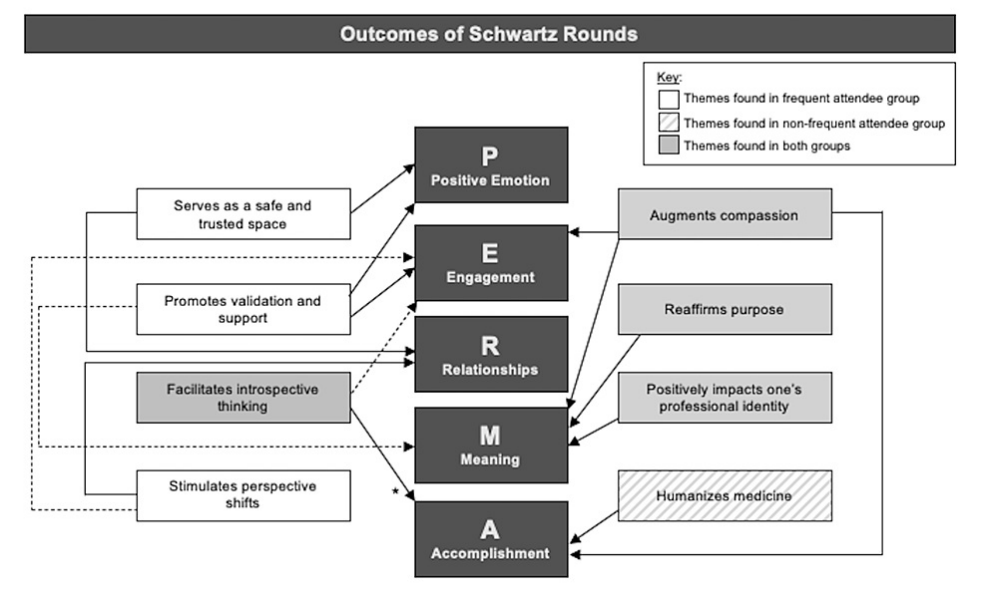
Focus Group Themes Aligned to PERMA Focus group themes from SR-specific questions and how they contribute to PERMA categories. The theme “no impact on resilience” is not represented in this figure. This theme existed for both frequent and non-frequent attendee groups and is categorized under “positive emotion.” For a theme to be represented within the frequent or infrequent group, ≥2 supporting quotations from attendees within their respective groups must have been identified. PERMA: Positive emotions, Engagement, Relationships, Meaning, Accomplishments; SR-Schwartz Rounds. * The theme facilitates introspective thinking was not categorized under “accomplishment” for the non-frequent attendee group but instead only for the frequent attendee group.

The participants were then asked to expound upon the overall impact of SR on their well-being. Here, we will present the nine emerging themes that stemmed from these discussions as they relate to the theory of PERMA (Figure 1), while also providing representative quotations (Table 3).

**Table 3:** Focus Group Themes, SR-Specific Questions, Direct Representative Quotations, and PERMA Categories FR: Frequent Schwartz Rounds attendee; NFR: Non-frequent Schwartz Rounds attendee; PERMA: Positive emotions, engagement, relationships, meaning, accomplishments; SR: Schwartz Rounds. *While quotations are provided from both groups to support this theme, this theme was only present in one group (either frequent or non-frequent attendee group). Please refer to Figure 1 to see which themes were represented in each group.

POSITIVE EMOTION	
Theme(s)	Do you think Schwartz Rounds have helped improve your well-being? Why or why not?
Promotes validation and support*	Frequent Attendee: “In Schwartz Rounds where we’re so isolated in the NICU…there were other people that felt the same way I did…You're not alone. We're not alone in this isolated box.” — FR1
	Non-Frequent Attendee: “It’s nice to sometimes unburden and feel like other people understand what you’re going through…it doesn’t make me feel as isolated and if you are able to share those experiences and have other people that feel the same way or even to offer a different perspective….” — NFR1
Theme(s)	Do you think Schwartz Rounds have helped you build resilience?
No impact on resilience	Frequent Attendee: “Like [Schwartz Rounds] may not on its own increase your ability to be compassionate or your ability to be resilient. But it does support what's there already. So whatever baseline of that you have, I think [Schwartz Rounds] does support and allow [resilience] to flourish…” — FR3
	Non-Frequent Attendee: “I don’t know about resilience. [Schwartz Rounds] is more of a discussion forum as opposed to a skill building. That’s maybe where I would differentiate.” — NFR2
ENGAGEMENT	
Theme(s)	Let us know how Schwartz Rounds have helped improve your engagement with your patients.
Facilitates introspective thinking	Frequent Attendee: “Because over there [in Schwartz Rounds] I have different disciplines sitting and talking about the case and giving feedback to me and the feelings and perceptions are very different. Not many people speak up when you have a situation like that but having a Schwartz Rounds and when you relax, and you are discussing about that scenario and the perceptions and feelings—that gives you an opportunity to incorporate it into your daily workflow.” — FR5
	Non-Frequent Attendee: “The last Schwartz Rounds for me was powerful ‘cause I think of myself as being aware of mental health issues. But the particular patient that we talked about—I had to catch myself and my biases…with that patient, I felt annoyed and bothered every day, but then going back and hearing the different perspectives, I’m like ‘oh, that was a lot for the family’ and ‘that was a lot for that person’… it’s nice to catch my biases…” — NFR7
Theme(s)	Do you think Schwartz Rounds have contributed to your ability to provide compassionate care?
Augments compassion	Frequent Attendee: “I think that Schwartz Rounds just I think hopefully supports the ability of people who are already bringing their compassion. Like it just gives them maybe a little validation that other people are thinking about the same things or just a space to talk about these things.” — FR3
	Non-Frequent Attendee: “Yes, I definitely think [Schwartz Rounds] helped to contribute… [Schwartz Rounds] definitely just is very eye-opening and has been such a great experience to have. And I think it definitely contributed to me being a more compassionate provider.” — NFR6
RELATIONSHIPS	
Theme(s)	Do you think Schwartz Rounds have helped build trust in your interprofessional community?
Serves as a safe and trusted space*	Frequent Attendee: “I think that by listening in [Schwartz Rounds’] sessions, it’s a very trusting-type environment…we’re all equal at that time, we’re all in an environment where we can speak about your experience and not feel concerned that it might come back to bite you in some way because the way they present it and the way they offer that open door for you. So, I figured it feels like that has created that safe space there.” — FR6
	Non-Frequent Attendee: “[Schwartz Rounds] is a safe space to talk about issues… It's more this overall feeling of [being] more close to the person, like you know them in a bit of a bigger way than just a colleague, even though you're not talking about it all the time. It's just the feeling that this is a trustworthy colleague.” — NFR5
Theme(s)	How do you feel like they have changed your engagement with colleagues?
Stimulates perspective shifts*	Frequent Attendee: “I think for me, just like, to learn about other people… you hear like all these stories and all of a sudden you look at people differently, right? Can't help yourself. And I think, maybe I got a little older, hopefully a little wiser, have a softer touch with people. Cause you have no idea what their morning's like. You know. Forget to mention what their month, or week, or year was like, you know. And what personal tragedies are going on in their home…”— FR4
	Non-Frequent Attendee: “I wouldn't say [Schwartz Rounds] has changed how I interact with [my colleagues], but maybe just how I perceive them.” — NFR3
MEANING	
Theme(s)	Do you think Schwartz Rounds have helped create purpose in your clinical care?
Reaffirms purpose	Frequent Attendee: “I think [Schwartz Rounds] validates my purpose more than creating. I think it validates my purpose and why I’m doing it or how I’m doing it… At first, I walked in, and I thought, ‘well, this really doesn’t have much to do with me. I know that some of my patients have psycho-social issues and stuff.’ But the more I was listening to it, the more I had this feeling of a purpose that I had to bring this information back to my team.” — FR6
	Non-Frequent Attendee: “I would say for me the purpose is already there. Um, I'm not sure that [Schwartz Rounds] has created purpose. Uhm, I would say that it has reaffirmed my purpose.” — NFR5
Theme(s)	Has Schwartz Rounds impacted any other aspect of your professional identity? If so, how?
Positively impacts professional identity	Frequent Attendee: “I think in the way I communicate… Like to bring up things a little bit more quickly or be a little bit more—I wouldn't say provocative—but, but if there's an issue, to bring it up in a way that it can be discussed rather than just sort of stewing over it in my own head. So, I think in that way, the communication that we model at Schwartz Rounds and that we try to achieve at Schwartz Rounds has been helpful to me outside of that.” — FR3
	Non-Frequent Attendee: “I think attending Schwartz Rounds and seeing what is out there has stimulated that aspect of my professional identity in that I really am now motivated to champion this sort of thing for particularly outpatient staff, who don't necessarily have the opportunity to come up to the hospital for [these] things.” — NFR5
ACCOMPLISHMENT	
Theme(s)	What do you feel like the mission in patient care is within your department or unit? How do you think Schwartz Rounds have contributed to that mission?
Humanizes medicine*	Frequent Attendee: “I think it has… the whole point of Schwartz Rounds, is to start talking about the things that aren't so black and white. This is not an M&M. You know, this is an emotional, personal ride and to address the humanity of what it is that we do. And I think it highlights, without being too esoteric, like the art of medicine.” — FR4
	Non-Frequent Attendee: “I think for me, [Schwartz Rounds] brings the humanism back in medicine… I really try to go into each patient encounter, treating them as a human and not like this number or this patient room or this… We’re always like ‘room 9, room 7.’ It’s always like I’m labelling the person or focusing on the system. [Schwartz Rounds] brings humanism back.” — NFR7

SR serving as a safe and trusted space

Frequent attendees were able to vent their frustrations and discuss hardships such as the loss of a patient. They perceived SR as a safe space to express their emotional experiences, with an emphasis on appreciation for being able to do this with their colleagues:

We’re all equal at that time. We’re all in an environment where we can speak about your experience and not feel concerned that it might come back to bite you in some way because of the way they present it and the way they offer that open door for you. (FR6)

I enjoy SR because a lot of the time, I feel the support that, yes, I’m doing it right. And there are other people, like-minded, that I can share that with. (FR7)

The forum also enabled frequent attendees to feel secure enough to express their vulnerabilities and even inadequacies as healthcare workers.

In contrast, non-frequent attendees described SR as a “safe environment” without much elaboration or scope in comparison to the more detailed narratives of frequent attendees. Notably, these contributions to the elements of Positive Emotions and Relationships were present in only the frequent attendee group.

SR promoting validation and support

SR helped this group of healthcare workers feel supported by enabling them to gain insight through others’ lived experiences. Through these conversations, frequent attendees were able to establish connections and identify support resources. Some responses from frequent attendees revealed the emotional impact of this dialogue: 

But like I said, in Schwartz Rounds where we’re so isolated in the NICU that I felt like there were other people that felt the same way I did. And then I learned about other resources… You’re not alone. We’re not alone in this isolated box. (FR1)

SR also helped validate healthcare workers’ perspectives on patient care through the sheer act of sharing stories with one another. Frequent attendees mainly recognized this theme and offered additional perspective on how SR impacts their professional identity.

I think it validates especially when I’m with such a group of people who really have a passion for what they do and care about what they… I’m happy to be part of this profession and this group that I’m working with because look at the impact that they are having just on each other about this one conversation and then listen to the stories of what they are doing in other places. (FR6)

Through SR, frequent attendees recognized their patient care work was greater than themselves and gained a larger appreciation for the importance of their roles. These responses helped the researchers identify the theme SR promotes validation and support only for the frequent attendee group, which highlights the elements of* Positive Emotion, Engagement, *and *Meaning.*

SR facilitating introspective thinking

SR promoted introspective thinking in multiple participants as they detailed how the forum provided them a space to discuss cases and receive real-time meaningful feedback. This helped both groups of attendees further navigate their judgments and knowledge about patient care:

I do work with a lot of high-risk patients, and their psychosocial issues sometimes just floor me. And sometimes, it’s in a way that I can’t relate but when you’re listening in SR and you hear many of the other stories or things or the way things are happening, you can just say is that a bias of mine? Is it just that I’m not relating? Where am I coming from? So, it makes you kind of have a little bit more of an introspective look on yourself. (FR6)

The last SR, for me was powerful ‘cause I think of myself as like being aware of mental health issues but the particular patient that we talked about, I had to catch myself and my biases. (NFR7)

The researchers recognized both groups were actively involved in meaningful introspective thinking which underlies aspects of *Engagement.*

Frequent attendees also understood the degree to which empathetic communication can be a challenge in a high-stress work environment. Through feedback from their colleagues in other departments, these participants gained new insight into how they can apply improved communication with patients in such settings. As such, the researchers recognized that the forum assisted the frequent attendee group in achieving specific goals within patient care, highlighting the element of *Accomplishment. *

SR stimulating perspective shifts

Frequent attendees found that the new perspective gained in SR helped to broaden their understanding of the patient’s experience and potentially enrich their clinical work:

We did a Schwartz Rounds a couple years back about a NICU baby that was sort of perceptually not gonna do well. [The baby] was in the NICU for a long time. And the staff kept saying, it’s been such a long time…. And somebody pointed out that for the parents this was the only six weeks they were ever gonna get to be with this child. And so, I think some of those perspective shifts that have come out of Schwartz Rounds have been huge. (FR3)

Perspective shifts also translated into interprofessional relationships, as SR helped individuals see different sides of their colleagues and created some degree of social connection. Some participants implied that it enabled them to empathize with each other more. The researchers established that perspective shifts were not present in the non-frequent attendee group as only one participant commented on this theme. For the frequent attendee group, this theme contributed to elements of *Engagement *and *Relationships.*

SR augmenting compassion

Providing compassionate care is part of the mission for many clinicians and helps them to derive meaning and purpose. Importantly, participants from both groups expressed that rather than creating compassion within providers, SR helped to augment the ability of healthcare providers to provide compassionate care by spending more time in the discussion of the psycho-emotional aspects of clinical care.

I think most of us in healthcare are compassionate people in general. I think healthcare self-selects for that. But, I do think that you do need to refill your tank, because you can get compassion fatigue… if you're feeling completely empty, I think patient interactions can be more problematic. And so, what I think SR does is it helps you fill your tank. (NFR5)

Based on other similar responses, researchers found this theme to contribute to* Engagement, Meaning, *and *Accomplishment *for both groups*.*

SR reaffirming purpose

There was sparse commentary on SR’s ability to generate purpose for both groups. Instead, participants felt that SR “validates purpose, not create purpose” (FR7) and since “the purpose is already there, it reaffirmed their purpose” (NFR1) as healthcare workers. This theme was found to contribute to the *Meaning *category.

SR positively impacting one’s professional identity

Professional identity was defined differently for individual participants, but SR was overall found to positively reaffirm aspects of it for both groups. Participants expressed that SR could break down barriers between healthcare workers and open new avenues of communication. SR also promotes aspects of patient advocacy through open and honest conversations among healthcare workers.

Maybe, in the way I communicate… if there's an issue, to bring it up in a way that it can be discussed rather than just sort of stewing over it in my own head. (FR3)

I think [SR] is helping me be a little bit of a better advocate [on things] that I used to just check off boxes of. (FR4)

Non-frequent participants reported that SR positively impacted aspects of their professional identity and helped them approach their patients with greater understanding, although providing briefer responses for this theme compared to those of the frequent participant group. This theme contributed to the category of *Meaning*.

SR humanizing medicine

Aspects of SR’s ability to humanize medicine were touched upon primarily by non-frequent attendees. Frequent participants did not discuss this theme except for one participant (Table 3). Non-frequent attendees revealed how SR helped humanize both patients and colleagues. This theme was found to relate to the *Accomplishment *category.

It does humanize-it's nice that you see [colleagues] a little different. You're like ‘oh, I never knew that about you.’ (NFR3)

It brings the humanism back in medicine… I really try to go into each patient encounter, treating them as a human and not like this number or this patient room. (NFR7)

No impact on resilience

Participants were asked if SR helped to build resilience. Both groups established that SR had no impact on resilience.

## Discussion

The objective of this study was to understand the impact of SR on the well-being of clinicians using the theory of PERMA and human flourishing. Previous studies have already identified SR as a tool that decreases psychological distress among healthcare providers [6,7]. Through qualitative exploration, this pilot study’s findings suggest a greater enrichment of flourishing as understood through the PERMA elements and demonstrate the deeper level of impact that SR has on clinician well-being.

Positive emotion

By providing safe spaces for open conversations and offering a well of validation and support through their own colleagues, SR was found to augment the experience of *Positive Emotions* in this group of healthcare workers. Interventional tools such as SR that promote positive emotions on a continual basis can have its own innate therapeutic benefit and improve one’s psychological well-being in the long term. According to the Broaden and Build Theory as described by Fredrickson [18], the experience of positive emotion is thought to expand the mind’s cognitive reserve and attention which can, in turn, improve one’s creativity, flexibility, and self-adaptive behaviors. In the healthcare setting, these attributes are essential to ensure the long-term health and longevity of the healthcare workforce. Importantly, as positive emotions may expand the individual’s attention, negative emotions such as loneliness, depression, and compassion fatigue can also narrow one’s attention and abilities to carry out decisive actions. Pfifferling and Gilley explained that compassion fatigue, a state of emotional exhaustion that is experienced by many healthcare workers, can lead to decreased workplace productivity, increased healthcare worker turnover rates, and a worsened quality of care [19]. Thus, strategies that promote the “broadening” process and experience of positive emotion are arguably crucial to healthcare workers’ sustenance.

Furthermore, prior research on SR by Adamson et al. demonstrates the importance of the “dose effect,” in which individuals who attend two or more SR compared to those who attend one demonstrated greater engagement in SR and thus gained more benefits [3]. This finding underscores the proposition that SR could offer clinicians a monthly (or bi-monthly) dose of *Positive Emotion*, which would help foster individual flourishing in the long term and cultivate a more enduring workforce.

Engagement

Maintaining a state of continuous *engagement* in providing compassionate care, as described in the Compassionate Care Flow model [20], can be difficult to do as a healthcare worker when considering the extent of emotional fatigue one can slowly accumulate over time. Thus, it is exceedingly important that forums such as SR exist to encourage thoughtful discussion about the difficulties of providing compassionate care all the time. This group of healthcare workers revealed that SR helped facilitate conversations that unveiled their own implicit biases in prior patient interactions which limited their ability to provide compassionate care to those patients. This introspective thinking enabled them to develop healthier perspectives about their patients and cultivate a more sustainable flow of inner compassion.

Moreover, by virtue of working in healthcare, clinicians are at greater risk for greater human suffering, compassion fatigue, and unresolved emotional trauma which was evident in many of the stories shared by the participants. SR appears to serve as a conduit for clinicians to process the psycho-emotional aspects of their traumatic experiences with their colleagues, and thereby offer some validation and support. As one participant worded it, SR can “refill your tank, because you can get compassion fatigue” (FR3), which epitomizes the importance of resources that help restore the well-being of healthcare providers and ultimately sustain a healthier flow in their compassionate care abilities.

Relationships

Collegial support can mitigate the effects of workplace burnout and loneliness [21]. For healthcare workers who work in isolation, meaningful social interaction becomes paramount to their well-being. Participants revealed that SR can serve as a pathway for the development of positive relationships with their colleagues. Moreover, Farr and Barker already established SR to have a positive impact on interprofessional relationships as 91% of survey-takers in their study expressed that SR would help them work better with colleagues [22]. This was supported by the study’s participants’ remarks which described SR as enabling them to relate to their colleagues on a human level and thus, building upon existing relationships. In our study, similar sentiments were expressed by frequent attendees as they gained new perspectives about their colleagues in conversations that were imbued with openness and vulnerability. Participants commented on how SR gave them the opportunity to see their colleagues differently which could potentially facilitate greater altruistic ties. SR’s ability to strengthen interprofessional relationships demonstrates the value of an open discussion forum. Any tool that enables clinicians to provide support to one another can also serve as a protective barrier against negative mental health outcomes.

Meaning

*Meaning* is a broad term that encompasses being a part of something greater than oneself and the pathway toward the fulfillment of something valuable [10]. Healthcare workers regard their own clinical work and professional identities to be meaningful and gratifying, as it is a way to channel a greater purpose in life. The researchers understood that SR had no way of creating purpose in healthcare workers, as participants expressed that comes from within. However, participants stated that SR reaffirmed the purpose of what they do, and reinvigorated and sometimes reformed their professional identities as it pertained to their communication styles, level of patient engagement, and ability to provide compassionate care. By augmenting these aspects of their professional identity, SR could be viewed as a tool that ties clinicians to the meaningfulness of their work, thus positively contributing to their well-being.

Accomplishment

SR appears to have an overall positive effect on participants’ job performance by enhancing aspects of their empathy, introspection, and reflective thinking which are all important skills to the work of a healthcare professional. Participants were actively utilizing these skills during SR as they considered the effects of their implicit biases and lack of perspective-taking in prior patient encounters. These internal conflicts were partially ameliorated once they broadened their understanding of their patients and re-evaluated their thoughts and actions through SR. In addition, participants expressed an innate desire to excel at their jobs and in their patient interactions, likely in part for their own sense of happiness and fulfillment. SR may serve as a conduit that clinicians can use to further progress in their professional goals.

Limitations

There were certain limitations of this study to be noted. This single-center study with an FG approach is limited to a small convenience sample of participants associated with our Children’s Hospital, and therefore, findings may not be generalizable to SR attendees at other institutions or from other specialties. FG participant responses may not represent the views of all SR attendees at our institution. Furthermore, due to scheduling conflicts, three FG sessions were conducted with a mix of FR and NFR SR attendees and one session had to be conducted as a 1-on-1 interview. Although the FG moderator was aware of FR versus NFR assignment of participants, to avoid interviewer bias and the domination of certain voices or perspectives, each participant was solicited to contribute a response to every question to ensure full opportunity for everyone to share their thoughts and experiences. Also, of the sixteen participants, two participants were not pediatric clinicians, but rather from other departments.

Moreover, while the PERMA diagram (Figure 1) showcases the respective themes and the group(s) to which they belong, the discussion portion of the paper was cautious not to delve extensively into the differences between the FR and NFR groups given the study’s small sample size and study design. Rather the purpose of showcasing the data through the delineation of FR and NFR participants was to simply offer a more nuanced refinement of the data. It is worth noting that although the COVID-19 pandemic’s impact on the outcomes of this qualitative study was not directly assessed, participants often commented on how the pandemic influenced their clinical work, patient engagement, colleague engagement, and emotional well-being (Table 2).

## Conclusions

This pilot study was the first to date to address how the impact of SR on clinicians can be understood through the lens of human flourishing. Human flourishing, a concept in positive psychology, is dynamic and embodies the everchanging and continuing development of humans as they strive for a fulfilled life. Through FG interviews, our study was able to delineate clinicians’ perspectives on SR and its ability to foster aspects of one’s flourishing which is primarily understood through aspects of PERMA. Our analysis suggests that SR not only serves as a group reflection tool, but it touches upon every aspect of clinician flourishing including their sense of *positive emotion*, sustained *engagement*, *relationship*-building, *meaning*-making, and *accomplishment* in the healthcare setting.

Attendees at our institution helped establish SR's impact on well-being as dynamic and multifaceted, supporting clinicians at both the individual and community levels. At the individual level, it cultivates a safe space for clinicians to heal and grow as they relieve themselves of their emotional burdens and receive validation. This effect can promote long-term sustenance and longevity within the workforce. Additionally, SR can facilitate introspective thinking, stimulate perspective shifts, augment compassion, reaffirm purpose, and positively impact the professional identity of clinicians. At the community level, SR facilitates an interprofessional dialogue that establishes meaningful connections and supports systems, which ultimately help clinicians thrive and flourish. The implications of these findings are vast, but primarily they offer institutions an effective interventional tool to promote elevated well-being in the workforce. Future studies should seek to investigate the significance of these findings on a broader, multi-institutional scale.

## Appendices

**Table 4:** Semi-Structured Interview Questions for Focus Groups

GENERAL QUESTIONS	
PROFESSIONAL	How do you feel about your clinical work? Is your clinical work gratifying/meaningful/joyful or draining and/or running on empty? How do you feel about your engagement with patients? How do you feel about your engagement with colleagues? Those who are in the same field as you? Those who are in other disciplines?
INDIVIDUAL	Overall, how do you feel about your emotional well-being? To what extent do you feel the things you do in your life are worthwhile?
SCHWARTZ ROUNDS QUESTIONS	
PATIENT CARE	Let us know how Schwartz Rounds has helped improve your engagement with your patients. How do you feel like they have changed your engagement with patients? What do you feel like the mission in patient care is within your department or unit? How do you think Schwartz Rounds have contributed to that mission? Do you think Schwartz Rounds have contributed to your ability to provide compassionate care?
INDIVIDUAL	Do you think Schwartz Rounds have helped improve your well-being? Why or why not? Do you think Schwartz Rounds have helped you build resilience? Do you think Schwartz Rounds have helped create purpose in your clinical care? Are empathy and compassion a part of your professional identity? Has Schwartz Rounds played a role in them? Has Schwartz Rounds impacted any other aspect of your professional identity? If so, how?
COMMUNITY	Do you think Schwartz Rounds have helped build trust in your interprofessional community? How do you feel like they have changed your engagement with colleagues? Do you think Schwartz Rounds have built satisfaction in your professional relationships? What is your shared purpose in your department or unit? How do you think Schwartz Rounds have contributed to that purpose?
